# Divergent roles of herbivory in eutrophying forests

**DOI:** 10.1038/s41467-022-35282-6

**Published:** 2022-12-22

**Authors:** Josiane Segar, Henrique M. Pereira, Lander Baeten, Markus Bernhardt-Römermann, Pieter De Frenne, Néstor Fernández, Frank S. Gilliam, Jonathan Lenoir, Adrienne Ortmann-Ajkai, Kris Verheyen, Donald Waller, Balázs Teleki, Jörg Brunet, Markéta Chudomelová, Guillaume Decocq, Thomas Dirnböck, Radim Hédl, Thilo Heinken, Bogdan Jaroszewicz, Martin Kopecký, Martin Macek, František Máliš, Tobias Naaf, Anna Orczewska, Kamila Reczynska, Wolfgang Schmidt, Jan Šebesta, Alina Stachurska-Swakoń, Tibor Standovár, Krzysztof Swierkosz, Ondřej Vild, Monika Wulf, Ingmar R. Staude

**Affiliations:** 1 German Centre for Integrative Biodiversity Research (iDiv) Halle-Jena Leipzig, Leipzig, Germany; 2 Institute of Biology, Martin Luther University Halle-Wittenberg, Halle (Saale), Germany; 3 CIBIO (Research Centre in Biodiversity and Genetic Resources)–InBIO (Research Network in Biodiversity and Evolutionary Biology), Universidade do Porto, Porto, Portugal; 4 Forest & Nature Lab, Ghent University, Gontrode, Belgium; 5 Institute of Ecology and Evolution, Friedrich Schiller University Jena, Jena, Germany; 6 Department of Biology, University of West Florida, Pensacola, FL USA; 7 UUMR CNRS 7058 “Ecologie et Dynamique des Systèmes Anthropisés” (EDYSAN), Université de Picardie Jules Verne, 1 rue des Louvels, F-80000 Amiens, France; 8 Institute of Biology, University of Pécs, Pécs, Hungary; 9 Department of Botany, University of Wisconsin-Madison, Madison, WI USA; 10 MTA-DE Biodiversity and Ecosystem Services Research Group, Egyetem tér 1, H-4032 Debrecen, Hungary; 11 Southern Swedish Forest Research Centre, Swedish University of Agricultural Sciences, Alnarp, Sweden; 12 Institute of Botany, Czech Academy of Sciences, Brno, Czech Republic; 13 Environment Agency Austria, Spittelauer Lände 5, 1090 Vienna, Austria; 14 Department of Botany, Faculty of Science, Palacký University in Olomouc, Olomouc, Czech Republic; 15 University of Potsdam, Institute of Biochemistry and Biology, Maulbeerallee 3, 14469 Potsdam, Germany; 16 Bialowieza Geobotanical Station, Faculty of Biology, University of Warsaw, Sportowa 19, 17-230 Bialowieza, Poland; 17 Institute of Botany of the Czech Academy of Sciences, Zámek 1, CZ-252 43 Pruhonice, Czech Republic; 18 Faculty of Forestry and Wood Sciences, Czech University of Life Sciences Prague, Kamýcká 129, CZ-165 21 Praha, 6 – Suchdol Czech Republic; 19 Faculty of Forestry, Technical University in Zvolen, Zvolen, Slovakia; 20 National Forest Centre, Zvolen, Slovakia; 21 Leibniz Centre for Agricultural Landscape Research (ZALF), Muencheberg, Germany; 22 Institute of Biology, Biotechnology and Environmental Protection, Faculty of Natural Sciences, University of Silesia, Katowice, Poland; 23 Department of Botany, Faculty of Biological Sciences, University of Wroclaw, Kanonia 6/8, PL-50-328 Wroclaw, Poland; 24 Department of Silviculture and Forest Ecology of the Temperate Zones, University of Göttingen, Göttingen, Germany; 25 Department of Forest Botany, Dendrology and Geobiocoenology, Faculty of Forestry and Wood Technology, Mendel University in Brno, Brno, Czech Republic; 26 Institute of Botany, Faculty of Biology, Jagiellonian University, Kraków, Poland; 27 Department of Plant Systematics, Ecology and Theoretical Biology, Institute of Biology, Loránd Eötvös University, Pázmány s. 1/C, H-1117 Budapest, Hungary; 28 Museum of Natural History, University of Wroclaw, Sienkiewicza 21, PL-50-335 Wroclaw, Poland; 29 Leibniz Centre for Agricultural Landscape Research (ZALF), Research Area 2, Müncheberg, Germany; 30 Institute of Biology, Leipzig University, Leipzig, Germany

**Keywords:** Forest ecology, Community ecology, Biodiversity, Conservation biology

## Abstract

Ungulate populations are increasing across Europe with important implications for forest plant communities. Concurrently, atmospheric nitrogen (N) deposition continues to eutrophicate forests, threatening many rare, often more nutrient-efficient, plant species. These pressures may critically interact to shape biodiversity as in grassland and tundra systems, yet any potential interactions in forests remain poorly understood. Here, we combined vegetation resurveys from 52 sites across 13 European countries to test how changes in ungulate herbivory and eutrophication drive long-term changes in forest understorey communities. Increases in herbivory were associated with elevated temporal species turnover, however, identities of winner and loser species depended on N levels. Under low levels of N-deposition, herbivory favored threatened and small-ranged species while reducing the proportion of non-native and nutrient-demanding species. Yet all these trends were reversed under high levels of N-deposition. Herbivores also reduced shrub cover, likely exacerbating N effects by increasing light levels in the understorey. Eutrophication levels may therefore determine whether herbivory acts as a catalyst for the “N time bomb” or as a conservation tool in temperate forests.

## Abstract (web summary)

Ungulate herbivory is an important driver of ecological change in forests. Here, the authors combine vegetation resurveys showing herbivory effects are highly dependent on soil eutrophication, promoting non-natives under high N-conditions, yet benefiting threatened species under low N-conditions.

## Introduction

Temperate forests represent globally important ecosystems both as habitats supporting a unique set of species and providing essential ecosystem services^1–4^. These ecosystems are threatened, however, by unprecedented forest dieback and loss of species diversity^5–7^. It is critical, therefore, to understand the processes that are beneficial or detrimental to forest functioning^8^. Herbivory by ungulates is an important driver of ecological change in forests and populations are broadly increasing across Europe. Yet their conservation role remains highly contended^9–17^. Effects of herbivory are often varied and highly context-dependent^10^, with studies rarely exploring interactions with other global change drivers. Herbivory and eutrophication have been shown to strongly interact and drive vegetation dynamics in grassland and tundra systems by mitigating light limitations and releasing low-stature, often threatened, species from competition^18,19^. However, this interaction is poorly understood in forests where nitrogen (N) deposition often continues to exceed critical loads^20–22^. Examining how herbivory interacts with N-deposition in forest plant communities is, therefore, key to making informed forest management and restoration decisions.

The second half of the 20th century witnessed the resurgence of many populations of wild grazer and browser species, increasing their density and range across European landscapes^23,24^. Several factors contributed to these trends, including restrictions on hunting, hunter desires for higher game densities, land abandonment, reduction of natural predators and deliberate reintroductions^25,26^. Human pressures have also acted to push some of these species from semi-open into closed forest systems^27^. Consequently, the majority of wild herbivory pressure now occurs in forests which can shape forest systems in different ways^23,28,29^. Herbivores can reduce understorey vegetation biomass and tree regeneration, compact soils and alter rates of nutrient cycling^30–32^. Through browsing and grazing lawns, herbivores can further create positive consumer-resource cycles that impact vegetation composition, enhance seed dispersal and structural heterogeneity^33,34^. Studies find highly heterogeneous, sometimes non-linear vegetation responses to herbivory^35^. Some plant species benefit, while others decline or disappear, in turn affecting composite indicators like plant cover and diversity^9–16^. The conservation effects of herbivory are yet more contentious. Some evidence suggests that herbivory can reduce threatened species^15^ while favouring non-natives^36^. Other studies find that herbivory suppresses competitive species, in turn favouring low-stature and threatened species^37–39^. Understanding the varying effects of herbivory is central to policy recommendations for forest and wildlife managers.

Concurrent with herbivore expansions, eutrophication of natural communities greatly increased over the last century largely in response to atmospheric N-deposition and other nutrients, as well as shifts in forest management^40^. This has led to the reordering of native woodland plant communities^6,22^. N-demanding species tend to be generalists with larger climatic and geographic ranges that are most competitive in areas with high N-loads^41^. Higher growth rates allow them to outcompete N-efficient species, many of which are of low-stature and/or with more restricted geographic ranges, traits typical of many rare and threatened species^41–43^. Nonetheless, experimental evidence of N-additions to forest understories appears less consistent than those observed in grasslands, with forest systems remaining more stable than predicted under increasing eutrophication^20–22,43–46^. Shifts towards a “high forest” management system over the last century have led to average increases in the biomass of tree and/or shrub layers across many temperate European forests^43,47,48^. The buffering capacity of canopies, accentuated by such a biomass increase, is hypothesized to attenuate the impact of N-deposition by reducing light availability to the understorey, generating time lags in vegetation responses^7,49–51^. The slow but pervasive effects of N-deposition have led some to label this threat a “N time bomb”^43^.

**Fig. 1:**
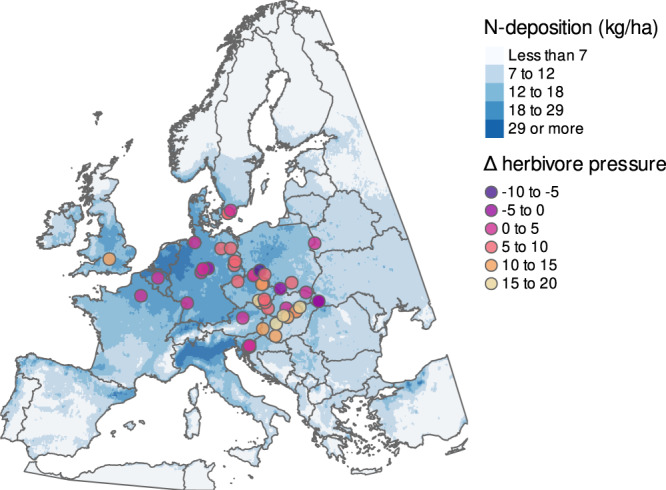
Spatial distribution of resurvey sites, N-deposition in the year 2000, and changes in (Δ) herbivory pressure across Central Europe. Our analysis spans 52 resurvey sites with inter-census time spans ranging from 10 to 64 years (median: 47.5 years). Color of points corresponds to the magnitude of change in site herbivore pressure between the baseline survey and resurvey (Supplementary Data file 1). Total nitrogen deposition (wet and dry, reduced and oxidized) is calculated using the EMEP database for the year 2000 and displayed across a color gradient of light to dark blue representing lowest to highest values at a spatial resolution of 10 km.

Given that large herbivores tend to reduce shrub and herb cover and height, they often increase light levels in the understorey (here, the herb layer)^52–54^, thereby potentially influencing N-effects and competition among plants^55,56^. Here we test three alternative hypotheses: (1) Increases in herbivory could alter the effects of N-deposition by mitigating light limitation and competitive effects on low-stature species as it does in grassland and tundra systems^18,19,57–59^; (2) as light regimes in forests differ greatly from grasslands, herbivory in forests might instead preferentially facilitate the spread of non-native, N-demanding species^60,61^, as these proliferate in N-enriched sites when light availability is high^62^; (3) herbivory does not interact with N-deposition as systematic increases in canopy cover^63^ attenuate any effect of herbivory on the shrub and understorey layers^56^. Our study leverages long-term vegetation data from 2928 resurveyed plots from 52 sites across seminatural temperate forests in Europe (median: 47.5 yrs between surveys; Fig. 1) to test these hypotheses. By quantifying the interactions between herbivory and N-deposition, we add to the growing debate about whether and under what conditions herbivory plays a role in contemporary forest management at times of unprecedented environmental change.

## Results

**Fig. 2:**
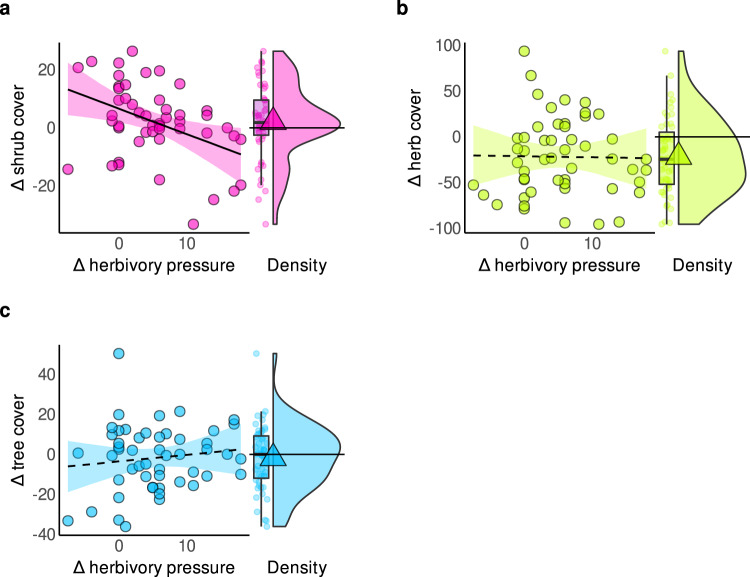
Higher herbivory decreased shrub layer cover, but not herb and tree layer cover. Relationships between changes in (Δ) herbivory pressure and **a** Δ shrub layer cover, **b** Δ herb layer cover, and **c** Δ tree layer cover. All models included inter-census time span, site area, and baseline herbivory as covariates. Note two sites lacked shrub and tree cover and one site also lacked herb cover data so that there were *n* = 50 and *n* = 51 independent resurvey sites for a, c, and b, respectively. Lines and ribbons represent the posterior mean line and the 95% credible interval. Dashed regression lines represent statistically unclear relationships. Frequency distributions (density, boxplot and points) of the respective response variables are displayed alongside. Boxplots bound the interquartile range (IQR) divided by the median and whiskers extend up to a maximum of 1.5 × IQR beyond the box. Triangles indicate the mean. Horizontal lines at zero indicate no change. Source data are provided as a Source Data file.

We found that, on average, shrub layer cover increased, herb layer cover decreased, and tree layer cover remained mostly constant over time in our forest sites. Increases in herbivory were clearly associated with declines in shrub layer cover (*β* = −0.42, σ = 0.17). However, it was statistically uncertain whether increases in herbivory were associated with changes in the herb and tree layer cover; both associations were neither strong nor very precise (*β* = −0.02, σ  = 0.17; *β* = 0.13, σ = 0.18 respectively; Fig. 2a–c and Supplementary Tables 3–5). Given that prior forest management may initiate different trajectories in these vegetation layers^38,56,64,65^, we tested for the role of historic, and recent changes in management. With the exception of a greater increase in tree cover at sites where management intensity had recently decreased, management did not clearly predict changes in vegetation cover, and the relationship between herbivory and shrub suppression persisted when management change was accounted for (Supplementary Fig. 1 and Supplementary Tables 6−11).

**Fig. 3:**
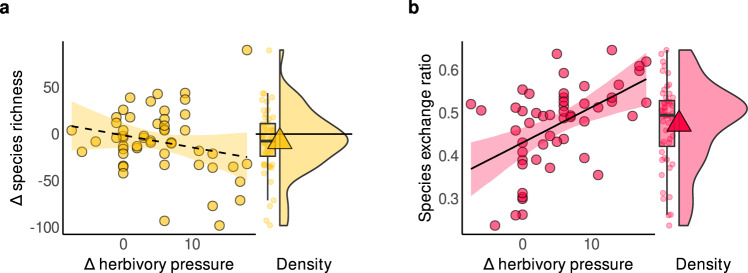
Herbivory increased temporal species turnover but was not clearly associated with changes in species richness. Relationships between change in (Δ) herbivory pressure and **a** Δ species richness (number of species) and **b** temporal species turnover (unitless) at a study site. All models included inter-census time span, site area, and baseline herbivory as covariates, with *n* = 52 independent resurvey sites. Lines and ribbons represent the posterior mean line and the 95% credible interval. Dashed lines represent statistically unclear relationships. Frequency distributions (density, boxplot and points) of the respective response variables are displayed alongside. Boxplots bound the interquartile range (IQR) divided by the median and whiskers extend up to a maximum of 1.5 × IQR beyond the box. Triangles indicate the mean. Horizontal lines at zero indicate no change. Source data are provided as a Source Data file.

Herb layer species richness tended to decrease over time at the site level, but it was statistically uncertain whether herbivory contributed to this trend; the posterior mean slope for this association was negative, yet the posterior distribution also indicated a 9% chance of a positive slope (*β* = −0.23, σ = 0.17; Fig. 3a and Supplementary Table 12). In contrast, greater increases in herbivory clearly elevated temporal species turnover in the herb layer (*β* = 0.52, σ = 0.14; Fig. 3b and Supplementary Table 13), accounting for the evident positive effect of inter-census time span on temporal turnover (*β* = 0.32, σ = 0.13; Supplementary Table 13). Moreover, this role of herbivory as a catalyst for community change was not confounded by changes in forest management (Supplementary Tables 14−15).

**Fig. 4:**
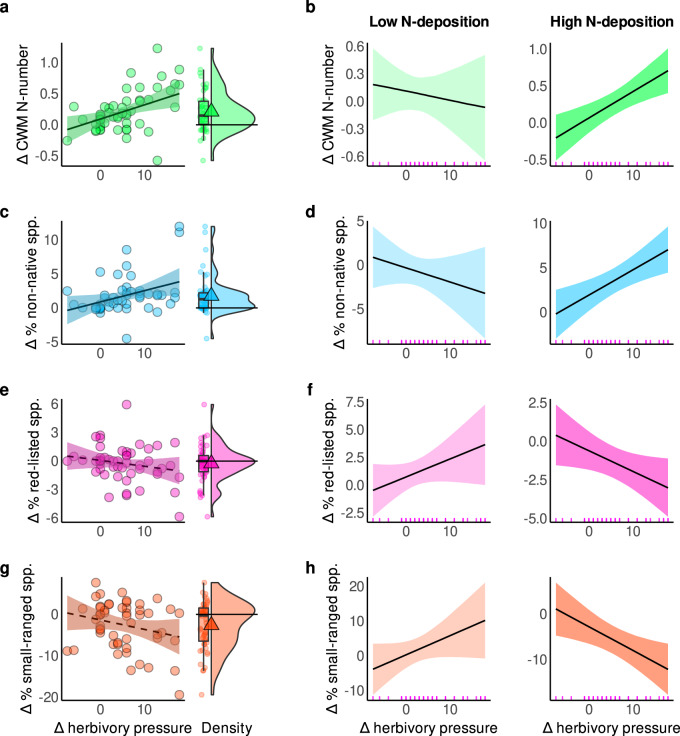
Herbivory effects depend on N-deposition levels. Relationships between change in (Δ) herbivory pressure and **a** Δ community-weighted mean N-number (CWM-N), **c** percentage change in (Δ %) non-native species, **e** Δ % red-listed species, and **g** Δ % small-ranged species. Frequency distributions (density, boxplot and points) of the respective response variables are displayed alongside. Boxplots bound the interquartile range (IQR) divided by the median and whiskers extend up to a maximum of 1.5 × IQR beyond the box. Triangles indicate the mean. Horizontal lines at zero indicate no change. Herbivory effects depend on N-deposition (**b, d, f**, **h**). Conditional effects of herbivory are depicted at the 10th (348 kg/ha; left) and 90th (1010 kg/ha; right) percentile of cumulative N-deposition in the data. There are *n* = 52 independent resurvey sites for all models. Lines and ribbons represent the posterior mean line and the 95% credible interval. Dashed lines represent statistically unclear relationships. Rugs in figure bottom in **b, d, f**, **h** depict the marginal distribution of the predictor. Cumulative N-deposition is calculated between the baseline and resurvey year per site. See Supplementary Fig. 3 and Supplementary Table 26−29 for model outputs of the effects of N-deposition alone. See Supplementary Fig. 4 for interaction effects on species richness change and exchange ratio. Source data are provided as a Source Data file.

By testing the separate effects of herbivory and N-deposition, we found that increases in herbivory shifted forest plant communities towards species with higher nutrient demands as inferred from increases in the community-weighted mean of species indicator values for nutrients (CWM-N) (*β* = 0.43, σ = 0.15, Fig. 4a and Supplementary Table 16). Similarly, greater increases in herbivory were associated with a higher proportion of non-native species (*β* = 0.37, σ = 0.17, Fig. 4c and Supplementary Table 17). Per contra, increases in herbivory tended to be negatively associated with the proportion of species classified as threatened in national Red Lists, or small-ranged species; however, these associations were uncertain, with 88 and 92% posterior probabilities for a negative slope, respectively (*β* = −0.19, σ = 0.17; *β* = −0.25, σ = 0.17; Fig. 4e, g and Supplementary Tables 18, 19). These ties between species turnover and herbivory again persisted when management change was accounted for, except for non-native species whose association became unclear, with a 97% probability of a positive slope (Supplementary Tables 20–23). Forest management itself was not associated with turnover, except for small-ranged species that declined with reductions in management intensity (Supplementary Table 23). To better understand the relationships between herbivory, shrub suppression and community composition, we tested how changes in shrub layer cover related to changes in non-native and N-demanding species. Increases in shrub layer cover reduced the proportion of non-native species (*β* = −0.37, σ = 0.14; Supplementary Fig. 2a and Supplementary Table 24). The association with CWM-N was also negative but, with a 93% posterior probability for a negative slope, statistically unclear (*β* = −0.19, σ = 0.13; Supplementary Fig. 2b and Supplementary Table 25). As expected from previous studies^41^, N-deposition reduced the proportion of threatened species (*β* = −0.46, σ = 0.21), but increased the proportion of non-native species (*β* = 0.56, σ = 0.20; Supplementary Fig. 3a, b and Supplementary Tables 26, 27). Higher cumulative N-deposition also tended to be associated with declines in small-ranged species (96% posterior probability for a negative slope) and increases in nitrophilous species (80% posterior probability for a positive slope), but these associations were uncertain as the 95% credible interval of the posterior mean slope included zero (Supplementary Fig. 3c, d and Supplementary Tables 28, 29).

Responses of all four variables to changes in herbivory not only varied but actually reversed direction between sites subject to low vs. high levels of cumulative N-deposition (Fig. 4b–h and Supplementary Tables 30–33). That is, the linear trends reported above masked consistent differences in how vegetation responses to herbivory depended on N-deposition. For example, increased herbivory was associated with higher proportions of threatened and small-ranged species at sites subject to low cumulative N-deposition, whilst reducing them at sites with high N-deposition (interaction: *β* = −0.38, σ = 0.19 and *β* = −0.51, σ = 0.18, respectively; Fig. 4f, h). Increased herbivory further reduced the proportion of non-native species at low N-deposition sites, whereas at high N-deposition sites, herbivory was associated with increases in non-native species (interaction: *β* = 0.36, σ = 0.17; Fig. 4d). Likewise, the association between herbivory and nitrophilous species reversed direction along the N-deposition gradient (Fig. 4b; *β* = 0.33, σ = 0.17). At sites with low cumulative N-deposition, increased herbivory was associated with lower CWM-N, while at sites with high cumulative N-deposition, increased herbivory yielded conspicuous increases in community N-numbers (Fig. 4b). Interaction effects for non-native and small-ranged species remained statistically certain when changes in forest management was accounted for, whereas effects became marginally uncertain for red-listed and nitrophilous species (97% posterior probability for a negative and positive slope respectively; Supplementary Tables 34–37). In sum, the role of herbivory in shaping forest understorey community composition appears to depend on levels of N-deposition.

## Discussion

Long-term data from 52 forest sites across Europe allowed us to assess the separate and combined effects of herbivory and eutrophication on changes in forest understorey composition. Specifically, we were able to test how shifts in herbivory and N-deposition interact to shape community composition. Herbivory did not mitigate the negative effects of eutrophication as found in grasslands^19,57,58^. Instead, it played divergent roles in forests that depended on historical accumulations of nitrogen inputs (Fig. 4). At high cumulative N-deposition, increased herbivory favored nitrophilous and non-native species while diminishing species of conservation concern. Conversely, at low cumulative N-deposition, herbivory reduced nitrophilous and non-native species while favoring species of conservation concern. These results suggest that herbivory can amplify the deleterious effects of terrestrial eutrophication in forests, despite providing conservation benefits when nutrient inputs are low. These contrasting, context-dependent roles of herbivory echo and may help to account for the reported heterogeneous effects of herbivory on forest community composition^26^. Our findings are further consistent with the hypothesis of a N time bomb in forests that may be triggered by disturbances that increase light availability, such as herbivory.

Herbivory may increase light availability and act on vegetation dynamics in several ways. Although shrub layer cover increased on average across the sites of our study, herbivory reduced it (Fig. 2a). This finding is consistent with other studies showing that herbivory can reduce the density and volume of woody vegetation^36,53,66^. Changes in tree layer cover, however, did not covary with changes in herbivory, and were furthermore not directional (Fig. 2c). While we see that recent declines in management intensity led to increases in canopy cover (Supplementary Fig. 1), thus impacting light availability, the relationships between herbivory and herb layer vegetation dynamics remained largely robust. Whilst herb layer cover and richness were not associated with herbivory, herbivory sharply accelerated species temporal turnover (Figs. 2b, 3a, b). As this catalyst role of herbivory persisted even after accounting for changes in management, we suggest this turnover may be partially driven indirectly by increased light availability following the suppression of shrub layer cover from herbivory (Fig. 2a and Supplementary Fig. 1). Turnover rates may further be directly affected by herbivores via browsing, grazing, rooting, fraying and stripping^9,36,67,68^ as well as zoochorous seed dispersal^32,69^, reducing some species and enhancing propagule pressure and colonization of others. Precisely which species benefit from these processes likely depends on additional factors, such as available ambient N.

In contrast to grassy systems where light is not a primary limiting factor, and eutrophication effects are fast-acting, N-deposition effects are hypothesized to be attenuated in the low-light conditions of forests^18,19,52,59,70^. This has led previous studies to posit an N time bomb, potentially catalyzed by disturbances, such as herbivory, that release light limitations and exacerbate N-effects^43^. This is different in grassy systems, where herbivory instead mitigates the biotic pressure and light limitation that results from eutrophication on resource-conservative species^59^. Such species in forests must, however, already be adapted to low-light conditions. Thus, herbivory may encourage resource-conservative, smaller-ranged and threatened species under low N-levels (Fig. 4, Supplementary Fig. 5, and Supplementary Tables 38, 39) by selectively feeding on more palatable species, thereby freeing up physical space. This is consistent with studies showing that herbivory favors low-stature herbs in the absence of eutrophication, many of which have lower N-demands^15,71^. Conversely, under elevated N-levels, selective feeding may not suffice to control N-demanding species, as these may be too strongly promoted by the convergent increase in N, light and disturbance. The twin drivers of high herbivory and N-deposition may therefore elicit the colonization and spread of nitrophilous and non-native species, as these often require nutrient-rich, open and disturbed sites^67^ (Fig. 4, Supplementary Fig. 5 and Supplementary Tables 38, 39). This is consistent with studies suggesting that herbivory directly promotes N-demanding, palatable species via browsing lawns in systems with elevated N-levels, but not where N-levels are low^34^. Finally, our results highlight that these herbivory-induced changes in species community composition are more often caused by species losses at high N-deposition, whereas species losses were offset by gains under low N-deposition (Supplementary Fig. 4).

Studies of herbivory effects typically rely on short-term comparisons involving artificial herbivore exclosure/enclosure sites, e.g., ref. 67. Our study instead leveraged data across a broad spatiotemporal scale to emphasize ecological realism with wild plant communities and free-roaming herbivores (Supplementary Data file 1). Nevertheless, our observational approach has limitations. Our approach cannot account for all potential confounding variables (or test for higher-order interactions among them). For example, although we did not find a statistically clear effect of site productivity here (Supplementary Tables 40–43), it would be useful to explore whether the interaction effects we observed would hold across larger productivity gradients. Furthermore, our dataset reflected the natural dominance of red, roe and fallow deer and wild boar species in Europe (Supplementary Data file 1). These species can have different feeding preferences, physiologies, and biomass requirements to the larger feeders such as bison and moose^72^ that were only present in a few sites. Our results are likely to be driven by these dominant species, making it essential to understand whether the observed relationships would hold should populations of other herbivores increase. Finally, our herbivore densities reflect expert local knowledge, subject to uncertainty and error, particularly for the baseline surveys. More precise experimental approaches will be essential to substantiate our findings and fully account for potential collinearities of key variables, such as management, N-deposition and herbivory; however, these may realistically not match the spatiotemporal scales of our study.

As ungulate herbivory broadly increases across Europe and N-deposition often continues to exceed critical loads^24,41^, our study suggests that herbivory and N-deposition can interact to shape forest ecosystems. The role of herbivory strongly depended on levels of forest eutrophication for all of the key indicators we examined. These interacting effects have important implications for conservation, and especially rewilding efforts that focus on the reintroduction of herbivores in forest settings. Despite recent efforts to curtail N-emissions, rates continue to exceed critical loads in many areas with potential legacy effects on communities in the future^45,73–75^. The ability of N-demanding and many non-native species to outcompete and displace rarer and more range-limited species of conservation concern are likely to amplify and sustain such legacy effects. Therefore, policies that effectively curtail N-emissions are essential for forest protection in the long run. Depending on our ability to do so, herbivory can act either to trigger the N time bomb or as a tool to bolster species of conservation concern in the future.

## Methods

### Database

We compiled baseline vegetation survey and resurvey data from 52 sites with associated herbivory data distributed across 13 European countries in the temperate deciduous forest biome (Fig. 1; see www.forestreplot.ugent.be and ref. 43 for inclusion criteria; Supplementary Tables 1, 2). These sites occur in historically continuously forested natural and seminatural forests that have not experienced any substantial change in land use (i.e., no stand-replacing disturbance) either prior to the baseline survey or between the two surveys (sensu ref. 76). Site areas ranged from 5.5 ha to 2.5 × 10^6^ ha (median: 2300 ha). At each site, researchers surveyed species in the herb, shrub and tree layer across 10 to 190 permanent or quasi-permanent plots per site (median: 50; total: 2928). Time intervals between the baseline survey and resurvey ranged from 10 to 64 years (median: 47.5 years). We accounted for changes in species taxonomy between surveys and sites by harmonizing species names following GBIF’s backbone taxonomy^77,78^. This prevented double-counting species or inferring inflated estimates of turnover. Altogether, our dataset contains 1257 species across all sites and time periods. Note, because we include new resurveys here and herbivore densities were not available for all surveys in the forestREplot database, our data comprise a different set of sites than previous forestREplot publications (e.g., 50 and 15% overlap with refs. 43,56).

### Explanatory variables

#### Herbivore pressure

We quantified ungulate herbivore pressure at the level of a study site for the baseline survey and resurvey time period based on expert assessment from each site’s dataset custodian in the forestREplot network^18^. Custodians provided density estimates using the best available information alongside expert knowledge of the site. Many of these densities have been used in previously published analyses (see also refs. 8, 15, 37, 43, 79). Density estimates incorporate one or more of the following sources; interviews (e.g., with local foresters, site managers, hunters, and national park administration), published and unpublished local data records, extrapolation of local/regional hunting statistics and/or direct animal count surveys. Herbivore densities were iteratively checked and revised twice by each dataset custodian and internally reviewed by the entire consortium. Custodians provided herbivore densities per species of ungulate as the number of individuals per 100 ha. This was then converted to an ordinal scale from 0 (no herbivores present) to 8 (>500 individuals per 100 ha), to account for a margin of error in the raw herbivory densities (Supplementary Data file 1). In total, there were 13 ungulate species across all sites, ranging from roe deer to European bison. We also considered wild boars as herbivore species: (1) because plant biomass comprises the majority (~90%) of their diet and they substantially impact plant regeneration (e.g., refs. 31, 80); and (2) because their feeding and rooting habits affect plant cover, diversity, height and regeneration and can have ecosystem-level effects^81^. We then summed these ordinal values across species at each site and time period, to reflect the overall herbivore pressure, following a similar approach as in refs. 8, 18, 82 (see Supplementary Data file 1 for all herbivore data and description of ordinal scale). We then also measured the equivalents of basal metabolic rates by multiplying the mean body mass of a species by its ordinal value, and summing across species per site. Mean body mass per species was taken from the Phylacine database^83^. As this indicator correlated highly with the above herbivore pressure index (Pearson’s *ρ* = 0.82; Supplementary Fig. 6a) and given an extreme outlier of one site (*ρ* = 0.92 when this outlier was removed, Supplementary Fig. 6b), we used the herbivore pressure index above in all analyses to avoid leverage. Temporal change in this index was calculated as the difference between the last resurvey and the baseline survey values per study site^18^, with change values ranging from −8 to 18 (Fig. 1 and Supplementary Data file 1).

#### N-deposition

We quantified total cumulative N-deposition using the EMEP database (https://emep.int/mscw/mscw_moddata.html), using cumulative wet and dry deposition of oxidized and reduced nitrogen^84^. We calculated the cumulative N-deposition between the baseline year and the year of the resurvey based on the methods described in ref. 8. First, we calculated N deposition between 1900 and the year of the baseline survey (N_t1_); second, we quantified the cumulative N deposition between 1900 and the resurvey (N_t2_); and third, we calculated the difference, N_t2_ – N_t1_, to quantify cumulative N deposition between surveys. Therefore, cumulative N-deposition per site will be influenced by the rate of deposition per year, as well as the length of the intercensal intervals (i.e., sites with lower yearly rates but long intervals may have similar values to sites with high yearly rates but shorter intervals). The values of cumulative N-deposition ranged from 130 to 1296 kg ha^−1^ (Supplementary Table 2 and Supplementary Fig. 7b).

#### Site productivity

To control for the potentially confounding influence of productivity on vegetation responses to herbivory^85,86^, we obtained local environmental data from the EuMedClim database^87^ on both the potential evapotranspiration and annual precipitation for each site averaged across the baseline and resurvey years. We then calculated the annual precipitation to potential evapotranspiration ratio (AP:PET) as a productivity proxy metric^88^. The EuMedClim database ranges from 1901 to 2014, so any sites that had been resurveyed since 2014 were given the 2014 value as their resurvey value. We calculated the average of the two time points. The AP:PET values ranged from 0.58 to 2.1 across sites.

#### Forest management

Changes in forest management during the inter-survey period can lead to changes in light regimes and confound the role of herbivory^38,56,64,65^. Therefore, we compiled data on recent changes (baseline to resurvey) in the management intensity of our forest sites (Supplementary Table 2). Management intensity has not changed at 79% of the sites and decreased at 21% of the sites between surveys. In addition, as past management practices can lead to legacy effects^56^, we also compiled information on historical management practices in the 1800s and categorized management as either high forest (HF) or coppice with standards (CWS), the two predominant silvicultural systems at the time (following the approach of refs. 56, 89). Fifty-two percent of sites were managed with HF, 27% with CWS, and 21% with a mixture of both in the 1800s. Management was described using expert testimony and historical site records^56^.

### Response variables

#### Herb, shrub, and tree (canopy) cover

We classified vegetation layers as follows: herb (all vascular plant species <1 m), shrub (1–7 m) and tree/canopy (>7 m). For each time period and layer, we quantified the total cover value at the site level. To do so, we summed species cover values in each plot per site (where species plot cover was estimated visually as the percent cover within a given plot). Plot totals were summed across plots and then divided by the total number of plots at a site. We quantified temporal changes in layer cover by subtracting the baseline cover from the resurvey cover^8,90^. Two sites lacked shrub cover data, with one of these sites also lacking herb cover data, leaving 50 and 51 sites available for study for these respective variables. Changes in herb layer cover ranged from −94 to 67%, changes in shrub layer cover ranged from −24 to 22%, and changes in tree layer cover ranged from −50 to 29%.

#### Species richness change and exchange ratio

Species richness change was calculated as the difference in the number of herb layer species at each site between the resurvey and baseline survey^8,90^. Herb layer species turnover was estimated using the richness-based species exchange ratio^91^ calculated at the site level as E = (S_imm_ + S_ext_)/S_tot_, where S_imm_ is the number of species gained at resurvey, S_ext_ is the number of species lost at resurvey and S_tot_ is the total number of unique species at baseline and resurvey. The range of change values for species richness and exchange ratios were −98 to 90 and 0.24 to 0.65, respectively.

#### Species of conservation concern

We identified species of conservation concern using two criteria: (1) Species listed as threatened in national Red Lists based on a recent database synthesizing national Red Lists across Europe^92^. That is, we determined the threat status of each species at a given site based on the respective national Red List of the country in which that site was located (based on IUCN threat classifications, see ref. 93). We then calculated the percentage of threatened species per site per survey period (baseline site mean = 2.2%, resurvey site mean = 1.9%). (2) Species that have small geographic range sizes. Our range size estimates are based on areas of occupancy (AOO, in km^2^) derived from point occurrence records in GBIF by ref. 93. We determined the lowest quintile of range size, which we classified as small-range. We then calculated the percentage of small-ranged species at each site and survey period and used differences between baseline and resurvey to quantify the temporal change (baseline site mean = 4.1%, resurvey site mean = 3.8%).

#### Non-native and nutrient-demanding species

We identified the non-native species present at each site using the Global Register of Introduced and Invasive Species (GRIIS; http://www.griis.org) and its designations of which species are non-native in each country. We then calculated the percentage of non-native species per site per survey period and the difference over time (baseline site mean = 3.0%, resurvey site mean = 4.8%). We estimated shifts in species’ N-demands using ecological indicator values (EIVs) compiled from ref. 94 (sci.muni.cz/botany/juice/ELLENB.TXT), filling data gaps with values from ref. 95. Coverage of N-numbers was 92% of species (1156 out of 1257 species). For each study site and survey period, we quantified the community-weighted mean N-number (CWM-N), weighted by baseline and resurvey occupancy per species per study site (i.e., the number of plots a species occupied during a given time period divided by the total number of plots at that site), and calculated the difference in CWM-N over time. Temporal changes in CWM-N ranged from −0.57 to 1.23 across sites.

#### Data analysis

We fitted Bayesian linear models using the “brms” package in R for all statistical analyses^96^. Data to reproduce the results of our study are available in the Supplementary Information and Source Data files. For all analyses, we ran four Markov chains. We set the default, weakly regularizing priors for all parameters. Convergence was assessed using the Gelman–Rubin statistics for each parameter (with values <1.01 taken to indicate adequate convergence) and visually inspecting trace plots^97^. The adequacy with which the models fit the data was examined using graphical posterior predictive checks. For all analyses, we used the 95% credible interval to determine statistical clarity^98^.

Response variables were changes in the shrub, herb, and tree layer cover, changes in species richness, species exchange ratio, and changes in threatened, small-ranged, non-native, and nitrophilous species. Our focal explanatory variable was the change in herbivore pressure over time. We controlled for baseline herbivory, as vegetation change is likely to unfold differently (for the same temporal change in herbivory) when starting herbivory was low vs. high^99–101^. We also accounted for total site area and inter-census time span in all models, as time and area affect the magnitude of vegetation change. Moreover, the inter-census time span mildly covaried with herbivory change (Pearson’s *ρ* = 0.25), and thus needed to be taken into account. Changes in herbivory, baseline herbivory, inter-census time span, and site area form the main variables for the models in our base results predicting vegetation change from herbivory change alone. We included additional covariates in supplemental models to test whether associations between the response variables and herbivory change held when accounting for forest management, tree cover change or site productivity. All main models held upon inclusion of site productivity (e.g., Supplementary Tables 40–43) and tree cover change (e.g., Supplementary Tables 44–47). Most models held upon inclusion of forest management and the three instances where this differed are reported in the main results. We also tested and confirmed that the effect of herbivory change was robust when baseline herbivory was excluded from the models (e.g., Supplementary Tables 48–51). Consequently, for the models testing the interaction effects between herbivory change and N-deposition on community composition, we only included site area and inter-census time span to reduce the risk of model overfitting (given that we only have 52 data points). We validated that the conditional association between cumulative N-deposition and changes in herbivory was statistically non-discernable from zero (Pearson’s *ρ* = 0.08; Supplementary Fig. 8), and hence these predictors did not covary in the model. Model syntaxes for the main models can be found on figshare at 10.6084/m9.figshare.21596844.

### Reporting summary

Further information on research design is available in the Nature Portfolio Reporting Summary linked to this article.

## Supplementary information

Supplementary Information

Description of Additional Supplementary Files

Reporting Summary

Supplementary Data 1

## Data Availability

The data generated in this study have been made available in the Supplementary Information. Source data for figures are provided with this paper. The underlying species composition data were available from forestreplot.ugent.be, but restrictions apply to the availability of these data, which were used under license for the current study and so are not publicly available. These data were, however, available from the authors upon request and with the permission of the forestREplot consortium. Source data are provided with this paper.

## Source data

Source Data files

## Code availability

The R code for all analyses is available on figshare at 10.6084/m9.figshare.21596844.
