# Supplementary material for: Divergent roles of herbivory in eutrophying forests
Source: Nat Commun. 2022 Dec 22;13:7837. doi: 10.1038/s41467-022-35282-6 (PMC9780218; doi:10.1038/s41467-022-35282-6)
Supplement: Supplementary file 2 — Description of Additional Supplementary Files [file 41467_2022_35282_MOESM2_ESM.pdf]

File Name: Supplementary Data 1
Description: This file provides the data for Supplementary Table 3, namely the herbivory information for each study site.
