# Supplementary material for: Divergent roles of herbivory in eutrophying forests
Source: Nat Commun. 2022 Dec 22;13:7837. doi: 10.1038/s41467-022-35282-6 (PMC9780218; doi:10.1038/s41467-022-35282-6)
Supplement: Supplementary file 3 — Reporting Summary [file 41467_2022_35282_MOESM3_ESM.pdf]

nature portfolio

Corresponding author(s): Josiane Segar

Last updated by author(s): 2022.11.14

Reporting Summary

Nature Portfolio wishes to improve the reproducibility of the work that we publish. This form provides structure for consistency and transparency in reporting. For further information on Nature Portfolio policies, see our Editorial Policies and the Editorial Policy Checklist.

Statistics

For all statistical analyses, confirm that the following items are present in the figure legend, table legend, main text, or Methods section.

n/a	Confirmed	
☐	☒	The exact sample size (*n*) for each experimental group/condition, given as a discrete number and unit of measurement
☐	☒	A statement on whether measurements were taken from distinct samples or whether the same sample was measured repeatedly
☐	☒	The statistical test(s) used AND whether they are one- or two-sided<br>*Only common tests should be described solely by name; describe more complex techniques in the Methods section.*
☐	☒	A description of all covariates tested
☐	☒	A description of any assumptions or corrections, such as tests of normality and adjustment for multiple comparisons
☐	☒	A full description of the statistical parameters including central tendency (e.g. means) or other basic estimates (e.g. regression coefficient) AND variation (e.g. standard deviation) or associated estimates of uncertainty (e.g. confidence intervals)
☐	☒	For null hypothesis testing, the test statistic (e.g. *F*, *t*, *r*) with confidence intervals, effect sizes, degrees of freedom and *P* value noted<br>*Give P values as exact values whenever suitable.*
☐	☒	For Bayesian analysis, information on the choice of priors and Markov chain Monte Carlo settings
☒	☐	For hierarchical and complex designs, identification of the appropriate level for tests and full reporting of outcomes
☐	☒	Estimates of effect sizes (e.g. Cohen's *d*, Pearson's *r*), indicating how they were calculated

Our web collection on statistics for biologists contains articles on many of the points above.

Software and code

Policy information about availability of computer code

Data collection	No software or code was used in the collection of community composition data. Data sources of site-level data are provided in the methods.
Data analysis	All Bayesian analyses were done in R Studio (v. 4.1.3) using the brms package (url: https://github.com/paul-buerkner/brms; v. 2.18.0). Model code and description is provided for all main analyses in the Supplementary Information. All data is provided in the Supplementary Information and corresponding Source Data files. All graphs are made with R Studio.

For manuscripts utilizing custom algorithms or software that are central to the research but not yet described in published literature, software must be made available to editors and reviewers. We strongly encourage code deposition in a community repository (e.g. GitHub). See the Nature Portfolio guidelines for submitting code & software for further information.

Data

Policy information about availability of data

All manuscripts must include a data availability statement. This statement should provide the following information, where applicable:

- Accession codes, unique identifiers, or web links for publicly available datasets
- A description of any restrictions on data availability
- For clinical datasets or third party data, please ensure that the statement adheres to our policy

The data generated in this study have been made available in the Supplementary Information. Source data for figures are provided with this paper. The R code for the paper is available on figshare (https://doi.org/10.6084/m9.figshare.21596844). The species composition data are available from forestreplot.ugent.be, but restrictions apply to the availability of these data, which were used under license for the current study and so are not publicly available. These data are, however,

available from the authors upon request and with the permission of the forestREplot consortium. We quantified total cumulative N-deposition using the EMEP database (https://emep.int/mscw/mscw_moddata.html). We obtained local environmental data from the EuMedClim database (https://doi.org/10.3389/fevo.2018.00031). We identified the non-native species present at each site using the Global Register of Introduced and Invasive Species (GRIIS; http://www.griis.org). Our plant species range sizes were determined from GBIF (https://www.gbif.org/). We got our threat status data from doi:10.1002/PPP3.10251.

Human research participants

Policy information about studies involving human research participants and Sex and Gender in Research.

Reporting on sex and gender	N/A
Population characteristics	N/A
Recruitment	N/A
Ethics oversight	N/A

Note that full information on the approval of the study protocol must also be provided in the manuscript.

Field-specific reporting

Please select the one below that is the best fit for your research. If you are not sure, read the appropriate sections before making your selection.

☐ Life sciences ☐ Behavioural & social sciences ☒ Ecological, evolutionary & environmental sciences

For a reference copy of the document with all sections, see nature.com/documents/nr-reporting-summary-flat.pdf

Ecological, evolutionary & environmental sciences study design

All studies must disclose on these points even when the disclosure is negative.

Study description	This study involves 52 resurvey studies of forest-floor plant communities across Europe's temperate forest biome. We analyse how site-level species communities change over time with respect to ungulate herbivory data interacting with cumulative inter-census Nitrogen deposition. We focus on the change in community-weighted mean traits and % changes of non-native and threatened species.
Research sample	The forestREplot database is the largest vegetation resurvey database for Europe's temperate forest biome to date. Studies in the forestREplot database are selected to match the data standards detailed in Verheyen et al .2017.<br>References:<br>Kris Verheyen et al., Combining Biodiversity Resurveys across Regions to Advance Global Change Research, BioScience, Volume 67, Issue 1, January 2017, Pages 73–83, https://doi.org/10.1093/biosci/biw150<br>This is supposed to represent a representative sample of the temperate forest vegetation biome across Europe. This sample was chosen because it provided data across a large spatio-temporal scale with naturally fluctuating associated herbivore densities.
Sampling strategy	Sample size was not predetermined but rather a maximum possible based on available resurvey studies. We were restricted to those sites that had accompanying herbivore data alongside the vegetation data provided by the data consortium.
Data collection	Community composition data were collected by several teams (see authorship contribution statement, teams are part of the forestREplot consortium). The data was collected using vegetation quadrants in several plots per site.
Timing and spatial scale	Baseline surveys were carried out between 1933 and 1999. The most recent resurveys were made between 1969 and 2017 generating time intervals of 10 - 64 years with a median of 47.5 years. Each site comprised 10 to 190 permanent or quasi-permanent plots per site (median: 50; total: 2928). The spatial scale of the data ranges from 5.5 to 2.5 x 10^6 ha (median: 2300 ha). Exact start and stop dates of sampling are unknown.
Data exclusions	No data were excluded.
Reproducibility	Our study is not experimental, but the large number of resurvey studies spread across Europe's temperate forest biome ensures good spatial cover. We have 52 resurvey sites that were resurveyed between 2 - 6 times depending on the site.
Randomization	Resurvey sites were selected to be located in semi-natural forests where no change in land use has taken place since the baseline survey. Within sites, plots in the baseline survey were allocated to provide a representative sample of the vegetation (phytosociological approach), corresponding to a stratified random sampling approach. We accounted for site differences by controlling for covariates (inter-census survey interval and site area).
Blinding	As our study is not experimental, blinding was not possible for data acquisition and analysis. Investigators were not blinded. Blinding during collection was not needed because conditions were well controlled. . Blinding during analysis is not necessary because the results are quantitative and did not require subjective judgment or interpretation. Blinding is not typically used in the field.

Did the study involve field work? ☐ Yes ☒ No

Reporting for specific materials, systems and methods

We require information from authors about some types of materials, experimental systems and methods used in many studies. Here, indicate whether each material, system or method listed is relevant to your study. If you are not sure if a list item applies to your research, read the appropriate section before selecting a response.

Materials & experimental systems

n/a	Involved in the study
☒	☐ Antibodies
☒	☐ Eukaryotic cell lines
☒	☐ Palaeontology and archaeology
☒	☐ Animals and other organisms
☒	☐ Clinical data
☒	☐ Dual use research of concern

Methods

n/a	Involved in the study
☒	☐ ChIP-seq
☒	☐ Flow cytometry
☒	☐ MRI-based neuroimaging
