# Supplementary material for: Divergent roles of herbivory in eutrophying forests
Source: Nat Commun. 2022 Dec 22;13:7837. doi: 10.1038/s41467-022-35282-6 (PMC9780218; doi:10.1038/s41467-022-35282-6)
Supplement: Supplementary file 1 — Supplementary Information [file 41467_2022_35282_MOESM1_ESM.pdf]

Supplementary Information for

Divergent roles of herbivory in eutrophying forests

Supplementary Figures

Supplementary Figure 1: *Vegetation cover changes under different forest management types.*

Supplementary Figure 2. *Association between shrub cover and nitrophilous, non-native spp.*

Supplementary Figure 3. *N-deposition effects on community composition.*

Supplementary Figure 4. *Species richness change and exchange ratio versus herbivory change in relation to N-deposition.*

Supplementary Figure 5. *Threatened and native spp. have lower N-numbers on average.*

Supplementary Figure 6. *Correlation between herbivory weighted with and without body mass.*

Supplementary Figure 7. *Histograms of herbivore pressure and N-deposition across sites.*

Supplementary Figure 8. *Conditional association between N-deposition and herbivore pressure.*

Supplementary Tables

Supplementary Table 1. *Study site info.*

Supplementary Table 2. *Dataset.*

Supplementary Table 3 - 11. *Model summaries for Figure 2: Herbivory and shrub, herb and tree layer cover changes.*

Supplementary Table 12 - 15. *Model summaries for Figure 3: Herbivory, species richness and temporal turnover.*

Supplementary Table 16 - 23. *Model summaries for Figure 4: Herbivory and community composition.*

Supplementary Table 24 - 25. *Model summaries for Supplementary Figure 2: Shrub cover versus non-native, nitrophilous spp.*

Supplementary Table 26 - 29. *Model summaries for Supplementary Figure 3: N-deposition and community composition.*

Supplementary Table 30 - 37. *Model summaries for Figure 4: Interaction between herbivory and N-deposition.*

Supplementary Table 38 - 39. *Model summaries for Supplementary Figure 4: Differences in N-numbers.*

Supplementary Table 40 - 43. *Model summaries for changes in herbivory versus community composition, accounting for productivity.*

Supplementary Table 44 - 47. *Model summaries for changes in herbivory versus community composition, accounting for tree cover*

Supplementary Table 48 - 51. *Model summaries for changes in herbivory versus community composition, excluding baseline herbivory.*

Supplementary Note

R code for all main analyses are available on figshare at: https://doi.org/10.6084/m9.figshare.21596844

Supplementary Figures

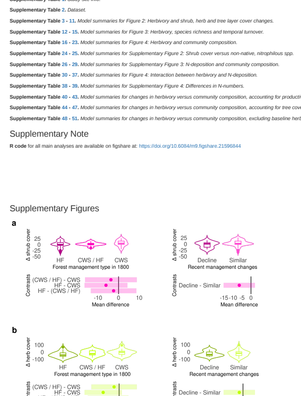

Supplementary Figure 1: **Relationship of (left) historical management practices and (right) recent management changes between changes in a, shrub cover; b, herb cover and c, tree cover.** The box and violin plots show that there is no association between forest management and changes in **a**, shrub cover or **b**, herb cover, either in the year 1800 (left) or due to recent management changes (resurvey - baseline). There is no association between historical forest management and **c**, changes in tree cover, however, there is an increase in tree cover in sites that experienced declines in management intensity between baseline and resurvey. Boxplots bound the interquartile range (IQR) divided by the median and whiskers extend up to a maximum of 1.5 x IQR beyond the box. Note that two sites lacked shrub and tree cover and one site also lacked herb cover data so that there were n=50 and n=51 independent resurvey sites for **a**, **c** and **b** respectively.

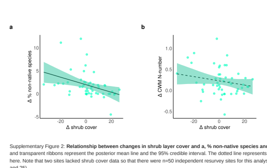

Supplementary Figure 2: **Relationship between changes in shrub layer cover and a, % non-native species and b, the CWM N-number**. Lines and transparent ribbons represent the posterior mean line and the 95% credible interval. The dotted line represents marginal non-significance here. Note that two sites lacked shrub cover data so that there were n=50 independent resurvey sites for this analysis (Supplementary Table 24 and 25).

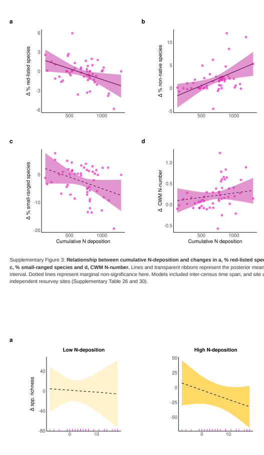

Supplementary Figure 3: **Relationship between cumulative N-deposition and changes in a, % red-listed species, b, % non-native species, c, % small-ranged species and d, CWM N-number.** Lines and transparent ribbons represent the posterior mean line and the 95% credible interval. Dotted lines represent marginal non-significance here. Models included inter-census time span, and site area as covariates, with n=52 independent resurvey sites (Supplementary Table 26 and 30).

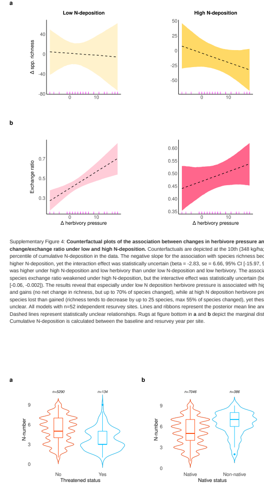

Supplementary Figure 4: **Counterfactual plots of the association between changes in herbivore pressure and species richness change/exchange ratio under low and high N-deposition.** Counterfactuals are depicted at the 10th (348 kg/ha; left) and 90th (1010 kg/ha; right) percentile of cumulative N-deposition in the data. The negative slope for the association with species richness became more pronounced under higher N-deposition, yet the interaction effect was statistically uncertain (beta = -2.83, se = 6.66, 95% CI [-15.97, 9.80]). Species exchange ratio was higher under high N-deposition and low herbivory than under low N-deposition and low herbivory. The association between herbivory and species exchange ratio weakened under high N-deposition, but the interactive effect was statistically uncertain (beta = -0.03, se = 0.02, 95% CI [-0.06, -0.002]). The results reveal that especially under low N deposition herbivore pressure is associated with high number of both species losses and gains (no net change in richness, but up to 70% of species changed), while at high N deposition herbivore pressure is associated with more species lost than gained (richness tends to decrease by up to 25 species, max 55% of species changed), yet these results remain statistically unclear. All models with n=52 independent resurvey sites. Lines and ribbons represent the posterior mean line and the 95% credible interval. Dashed lines represent statistically unclear relationships. Rugs at figure bottom in **a** and **b** depict the marginal distribution of the predictor. Cumulative N-deposition is calculated between the baseline and resurvey year per site.

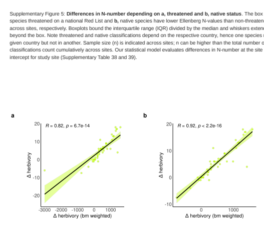

Supplementary Figure 5: **Differences in N-number depending on a, threatened and b, native status**. The box and violin plots show that **a**, species threatened on a national Red List and **b**, native species have lower Ellenberg N-values than non-threatened and non-native species across sites, respectively. Boxplots bound the interquartile range (IQR) divided by the median and whiskers extend up to a maximum of 1.5 x IQR beyond the box. Note threatened and native classifications depend on the respective country, hence one species may be native / threatened in a given country but not in another. Sample size (n) is indicated across sites; n can be higher than the total number of species in our data set as classifications count cumulatively across sites. Our statistical model evaluates differences in N-number at the site level by including a random intercept for study site (Supplementary Table 38 and 39).

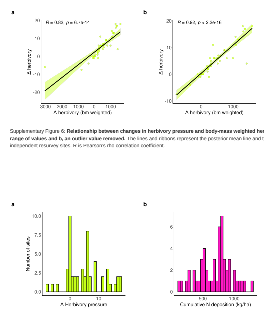

Supplementary Figure 6: **Relationship between changes in herbivory pressure and body-mass weighted herbivory pressure with a, the full range of values and b, an outlier value removed.** The lines and ribbons represent the posterior mean line and the 95% credible interval. n=52 independent resurvey sites. R is Pearson's rho correlation coefficient.

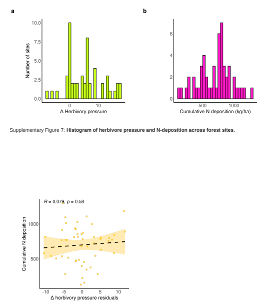

Supplementary Figure 7: **Histogram of herbivore pressure and N-deposition across forest sites.**

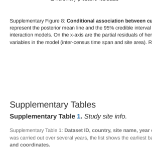

Supplementary Figure 8: **Conditional association between cumulative N-deposition and changes in herbivore pressure.** The line and ribbon represent the posterior mean line and the 95% credible interval for the conditional association between N-deposition and herbivory change in the interaction models. On the x-axis are the partial residuals of herbivore pressure change, namely the variation left unexplained by the other variables in the model (inter-census time span and site area). R is Pearson's rho correlation coefficient, p is the p-value.

Supplementary Tables

Supplementary Table 1. *Study site info.*

Supplementary Table 1: **Dataset ID, country, site name, year of the baseline survey and resurvey** [when one survey (baseline or resurvey) was carried out over several years, the list shows the earliest baseline survey and the latest resurvey in which all vegetation layers were sampled] **and coordinates.**

Dataset ID	Country	Site name	Baseline yr	Resurvey yr	Lat	Long
1	Belgium	Meerdaalwoud	1954	2000	50.799	4.708
2	Sweden	Dalby	1935	1976	55.692	13.330
3	Sweden	Dalby	1976	2002	55.692	13.330
4	Germany	Elbe-Weser	1986	2008	53.552	8.984
5	Czech Republic	Děvín Wood	1953	2002	48.867	16.630
6	Czech Republic	Milovice Wood	1953	2006	48.838	16.690
7	Czech Republic	Rychlebské hory Mts.	1942	1998	50.267	17.083
8	United Kingdom	Wytham Woods	1974	1999	51.773	-1.334
9	Germany	Göttingen, SFB	1980	2001	51.530	10.048
10	Germany	Göttingen, Carici-Fagetum	1960	2011	51.333	9.820
11	Germany	Göttingen, Hordelymo-Fagetum	1960	2009	51.556	10.019
12	Austria	Zöbelboden	1993	2005	46.840	14.440
13	Hungary	Heves	1989	2008	47.988	20.501
14	Germany	Brandenburg	1962	2012	52.059	13.857
15	Slovakia	Slovakia, South-West	1966	2007	48.398	17.341
16	Slovakia	Slovakia, Central	1964	2005	48.258	19.378
17	Slovakia	Slovakia, North-East	1965	2006	49.215	21.850
18	Czech Republic	České Středohoří	1965	2012	50.586	14.116
19	Czech Republic	Krumlov Wood	1964	2012	49.053	16.384
20	Czech Republic	Hodonínská Dúbrava	1965	2012	48.882	17.104
21	Czech Republic	Ždánice Wood	1959	2012	49.099	17.030
22	Poland	Białowieża	1966	2012	52.700	23.870
23	Sweden	Skåne	1983	2014	55.881	13.719
24	Hungary	Zselic	1958	2018	46.295	17.861
25	Germany	Göttingen, Hünstollen	1992	2002	51.578	10.047
26	Poland	Bazaltowa Mt	1992	2010	51.008	16.132
27	Poland	Buki Sudeckie beech forest	1990	2014	50.943	16.028
28	Poland	Trzebnickie Hills	1962	2011	51.262	16.816
29	Germany	Prignitz	1954	2014	53.080	12.280
30	Germany	Brandenburg Nord	1963	2014	53.064	13.471
31	Germany	Brandenburg Süd	1960	2014	51.792	13.801
32	Germany	Unteres Spreewald-Randgebiet	1965	2010	52.088	13.934
33	Slovenia	Strmec	1983	2015	45.622	14.819
34	Slovenia	Rajhenavski Rog	1983	2015	45.663	15.012
35	Slovenia	Pecka	1983	2015	45.755	14.999
36	France	Compiègne forest	1970	2015	49.364	2.887
37	Hungary	Bakony és Gerecse	1955	2015	47.202	18.075
38	Hungary	Bükkalja és Dél-Cserehát	1953	2014	47.909	20.399
39	Hungary	Gödöllői-dombság	1950	2014	47.589	19.396
40	Hungary	Mátra-Bükk-Zemplén	1958	2015	48.194	20.903
41	Hungary	Őrség	1954	2014	46.916	16.567
42	Hungary	Visegrádi-hegység	1953	2015	47.730	18.963
43	Germany	Großer Staufenberg	1988	1998	51.625	10.636
44	Germany	Riedried	1986	2018	49.033	8.230
45	Czech Republic	Śnieżnik Massif	1955	2013	50.231	16.917
46	Germany	Göttinger Wald (Fliehburgen)	1955	2015	51.576	10.008
47	Poland	N-E Puszcza Niepołomicka	1966	2019	50.093	20.373
48	Ukraine	Zakarpatska oblast	1935	1997	48.400	23.100
49	Ukraine	Zakarpatska oblast	1935	1997	48.400	23.100
50	Ukraine	Zakarpatska oblast	1935	1997	48.400	23.100
51	Poland	Riparian forests of the Oder Valley (near Wrocław)	1959	2019	50.930	17.350
52	Poland	Dolina Wapienicy (Wapienica Valley)	1991	2020	49.757	18.981

Supplementary Table 2. *Dataset.*

Supplementary Table 2: **Data table with key variables.** Shown here is the dataset ID; HP = change in herbivore pressure; Cum N-dep = cumulative N-deposition; RL% = % change in red list species/; Non-native % = % change in Non-native species; SR = change in species richness; herb cover; shrub cover; CWM-N = change in community weighted mean N value; Man 1800 = management in year 1800 (CWS = coppicing with standards; HF = high forest), Mgmt change = management change between baseline and resurvey.

Dataset ID	Δ HP	Cum N-dep	Δ RL %	Δ Non-native %	Δ SR	Δ Herb cover	Δ Shrub cover	Δ CWM-N	Man 1800	Mgmt change
1	0.0	1295.51	0.00	5.29	-4	-66.67	-11.90	0.14	CWS	Decline
2	6.0	544.46	5.94	2.20	-21	-14.14	-17.88	0.18	HF	Similar
3	6.0	630.31	-3.61	3.06	5	-46.96	-3.90	-0.07	HF	Similar
4	0.0	503.80	-0.31	0.54	26	-78.76	-12.73	-0.03	CWS / HF	Similar
5	6.0	1107.00	-3.45	-0.10	-93	-56.30	0.52	0.59	CWS	Decline
6	18.0	1185.39	-5.85	11.13	-32	-36.41	-19.72	0.89	CWS	Decline
7	6.0	1028.09	0.59	0.59	-33	-94.05	1.44	-0.22	HF	Similar
8	11.0	583.97	1.29	0.00	-18	24.87	NA	-0.11	CWS	Similar
9	0.0	456.89	0.03	0.00	-2	93.60	18.02	0.06	CWS	Similar
10	2.0	1095.38	-3.82	0.90	-15	46.47	26.39	0.27	CWS	Similar
11	2.0	1011.71	0.15	0.15	-13	10.86	8.07	0.20	CWS	Similar
12	0.0	223.66	1.53	0.00	4	-46.84	0.01	0.13	HF	Similar
13	0.0	344.98	-0.83	0.00	-34	NA	NA	0.17	HF	Similar
14	9.0	935.83	1.76	2.13	21	37.56	2.19	0.02	CWS / HF	Similar
15	7.0	783.12	0.83	2.40	-9	-3.06	3.50	0.22	CWS / HF	Similar
16	3.0	773.06	-0.62	0.67	-4	-3.90	5.17	0.30	CWS	Similar
17	3.0	770.90	0.00	1.52	-2	-24.73	-4.75	0.13	CWS / HF	Similar
18	7.0	874.61	-1.32	4.74	-10	-34.42	6.31	0.83	CWS / HF	Decline
19	18.0	890.17	-1.70	11.99	90	-24.56	-3.97	0.65	CWS	Similar
20	6.0	854.62	0.96	1.90	25	14.36	9.57	0.15	CWS / HF	Similar
21	6.0	923.88	-2.07	8.57	4	-51.24	0.73	0.38	CWS / HF	Similar
22	4.0	812.93	0.00	0.00	-7	-3.89	19.05	0.14	HF	Similar
23	4.0	516.31	-0.60	4.94	-9	-23.27	-1.55	0.11	CWS / HF	Similar
24	11.0	998.39	0.00	2.04	-33	-95.54	-33.20	0.40	HF	Similar
25	0.0	157.96	0.10	-0.73	-13	-15.31	14.21	-0.02	CWS	Similar
26	5.0	281.30	1.07	1.07	43	13.88	0.87	0.28	CWS	Similar
27	4.0	375.06	-1.64	1.12	35	31.91	4.12	-0.08	HF	Decline
28	-7.5	815.47	0.00	2.49	4	-52.73	-14.28	-0.26	HF	Decline
29	5.0	914.52	-0.70	2.61	11	-47.45	-1.32	0.18	CWS / HF	Similar
30	9.0	817.16	-3.14	1.76	4	22.67	15.14	0.40	CWS / HF	Decline
31	9.0	857.72	-0.44	1.52	44	26.20	4.45	0.60	CWS / HF	Decline
32	9.0	725.61	-0.83	2.50	37	-10.14	-0.28	0.38	HF	Similar
33	0.0	485.57	2.71	0.00	-35	-37.86	0.59	0.08	HF	Similar
34	0.0	474.97	2.41	0.00	-16	-46.00	13.56	-0.08	HF	Similar
35	0.0	467.00	0.00	0.00	-21	-75.62	9.51	-0.08	HF	Similar
36	1.0	677.73	0.37	1.46	39	66.96	10.13	0.29	HF	Similar
37	17.0	842.89	1.94	2.26	-51	-60.98	-21.79	0.66	HF	Decline
38	14.0	805.98	-0.79	1.89	-98	-93.10	-24.73	0.78	HF	Similar
39	13.0	814.59	-1.12	2.04	-21	-24.21	5.87	0.60	HF	Decline
40	17.0	774.21	-3.32	1.52	-71	-49.48	-2.77	0.28	HF	Similar
41	13.0	784.58	-1.36	1.84	-77	-56.74	-1.79	1.23	HF	Similar
42	16.0	800.16	-0.48	0.60	-35	-35.65	0.00	0.55	HF	Decline
43	-1.0	129.69	2.56	0.12	-4	-1.41	2.37	-0.10	CWS	Similar
44	1.0	399.87	-1.43	1.85	18	-60.40	14.81	0.03	CWS	Similar
45	13.0	753.30	1.64	-0.60	-36	-2.41	4.32	-0.57	HF	Similar
46	0.0	768.20	0.34	-0.18	-42	-0.73	22.34	0.09	CWS	Similar
47	6.0	683.00	-0.18	-4.51	16	40.37	19.62	-0.08	HF	Similar
48	-6.0	581.37	0.48	0.48	-19	-63.55	20.75	0.29	HF	Similar
49	-4.0	541.78	0.10	0.05	-8	-74.17	22.85	0.08	HF	Similar
50	-1.0	537.33	0.00	0.00	18	-28.19	4.26	-0.09	HF	Similar
51	6.0	677.41	0.58	6.19	9	11.22	-0.27	0.03	HF	Similar
52	-1.0	282.02	-1.14	-0.21	12	-13.08	-13.09	0.02	HF	Similar

Supplementary Table 3 - 11. *Model summaries for Figure 2: Herbivory and shrub, herb and tree layer cover changes.*

Supplementary Table 3: **Summary of the model testing the association between changes in herbivory and shrub cover (Figure 2a).** Parameter estimates, their standard error (SE) and 95% credible interval (CI). Both the response and predictor variables were scaled.

Predictor	Slope estimate	SE	lower 95% CI	upper 95% CI
Intercept	-0.01	0.14	-0.28	0.25
Herbivore pressure	-0.42	0.17	-0.76	-0.10
Baseline herbivory	0.14	0.15	-0.17	0.42
Time span	0.14	0.16	-0.17	0.45
Site area (log)	0.00	0.16	-0.31	0.33

Supplementary Table 4: **Summary of the model testing the association between changes in herbivory and herb cover (Figure 2b).** Parameter estimates, their standard error (SE) and 95% credible interval (CI). Both the response and predictor variables were scaled.

Predictor	Slope estimate	SE	lower 95% CI	upper 95% CI
Intercept	0.01	0.14	-0.27	0.28
Herbivore pressure	-0.02	0.17	-0.37	0.32
Baseline herbivory	-0.09	0.16	-0.41	0.22
Time span	-0.27	0.17	-0.59	0.06
Site area (log)	0.12	0.17	-0.23	0.46

Supplementary Table 5: **Summary of the model testing the association between changes in herbivory and tree cover (Figure 2c).** Parameter estimates, their standard error (SE) and 95% credible interval (CI). Both the response and predictor variables were scaled.

Predictor	Slope estimate	SE	lower 95% CI	upper 95% CI
Intercept	-0.01	0.14	-0.30	0.28
Herbivore pressure	0.13	0.18	-0.22	0.47
Baseline herbivory	0.12	0.17	-0.20	0.44
Time span	0.09	0.16	-0.24	0.41
Site area (log)	0.05	0.17	-0.29	0.38

Supplementary Table 6: **Summary of model testing the association between changes in herbivory and shrub layer cover, after accounting for forest management change.** Parameter estimates, their standard error (SE) and 95% credible interval (CI). Both the response and predictor variables were scaled.

Predictor	Slope estimate	SE	lower 95% CI	upper 95% CI
Intercept	0.061	0.154	-0.243	0.368
Herbivore pressure	-0.379	0.176	-0.725	-0.029
Baseline herbivory	0.168	0.153	-0.136	0.469
Time span	0.160	0.161	-0.148	0.481
Site area (log)	0.003	0.157	-0.306	0.306
Mgmt change:decline (delta)	-0.364	0.348	-1.037	0.318

Supplementary Table 7: **Summary of model testing the association between changes in herbivory and shrub layer cover, after accounting for historical forest management.** Parameter estimates, their standard error (SE) and 95% credible interval (CI). Both the response and predictor variables were scaled.

Predictor	Slope estimate	SE	lower 95% CI	upper 95% CI
Intercept	-0.254	0.218	-0.684	0.179
Herbivore pressure	-0.385	0.177	-0.738	-0.034
Baseline herbivory	0.143	0.155	-0.156	0.450
Time span	0.223	0.169	-0.107	0.555
Site area (log)	-0.121	0.235	-0.595	0.335
Mgmt: CWS/HF	0.472	0.508	-0.538	1.467
Mgmt: CWS	0.500	0.347	-0.168	1.169

Supplementary Table 8: **Summary of model testing the association between changes in herbivory and herb layer cover, after accounting for forest management change.** Parameter estimates, their standard error (SE) and 95% credible interval (CI). Both the response and predictor variables were scaled.

Predictor	Slope estimate	SE	lower 95% CI	upper 95% CI
Intercept	0.006	0.171	-0.330	0.341
Herbivore pressure	-0.017	0.181	-0.384	0.329
Baseline herbivory	-0.092	0.167	-0.428	0.239
Time span	-0.265	0.169	-0.603	0.064
Site area (log)	0.115	0.178	-0.227	0.461
Mgmt change:decline (delta)	0.000	0.370	-0.709	0.711

Supplementary Table 9: **Summary of model testing the association between changes in herbivory and herb layer cover, after accounting for historical forest management.** Parameter estimates, their standard error (SE) and 95% credible interval (CI). Both the response and predictor variables were scaled.

Predictor	Slope estimate	SE	lower 95% CI	upper 95% CI
Intercept	-0.196	0.224	-0.627	0.245
Herbivore pressure	-0.023	0.190	-0.407	0.346
Baseline herbivory	-0.088	0.161	-0.398	0.239
Time span	-0.177	0.180	-0.532	0.164
Site area (log)	0.085	0.251	-0.418	0.565
Mgmt: CWS/HF	0.246	0.552	-0.821	1.360
Mgmt: CWS	0.545	0.373	-0.210	1.293

Supplementary Table 10: **Summary of model testing the association between changes in herbivory and tree layer cover, after accounting for forest management change.** Parameter estimates, their standard error (SE) and 95% credible interval (CI). Both the response and predictor variables were scaled.

Predictor	Slope estimate	SE	lower 95% CI	upper 95% CI
Intercept	-0.152	0.168	-0.485	0.177
Herbivore pressure	0.059	0.178	-0.291	0.408
Baseline herbivory	0.055	0.166	-0.269	0.383
Time span	0.044	0.163	-0.279	0.354
Site area (log)	0.033	0.172	-0.316	0.369
Mgmt change:decline (delta)	0.678	0.373	-0.033	1.410

Supplementary Table 11: **Summary of model testing the association between changes in herbivory and tree layer cover, after accounting for historical forest management.** Parameter estimates, their standard error (SE) and 95% credible interval (CI). Both the response and predictor variables were scaled.

Predictor	Slope estimate	SE	lower 95% CI	upper 95% CI
Intercept	0.011	0.230	-0.443	0.459
Herbivore pressure	0.025	0.194	-0.338	0.418
Baseline herbivory	0.076	0.173	-0.253	0.411
Time span	0.105	0.180	-0.252	0.456
Site area (log)	0.282	0.254	-0.226	0.788
Mgmt: CWS/HF	-0.572	0.566	-1.683	0.534
Mgmt: CWS	0.354	0.366	-0.379	1.087

Supplementary Table 12 - 15. *Model summaries for Figure 3: Herbivory, species richness and temporal turnover.*

Supplementary Table 12: **Summary of the model testing the association between changes in herbivory and spp. richness (Figure 3a).** Parameter estimates, their standard error (SE) and 95% credible interval (CI). Both the response and predictor variables were scaled.

Predictor	Slope estimate	SE	lower 95% CI	upper 95% CI
Intercept	0.00	0.14	-0.27	0.27
Herbivore pressure	-0.23	0.17	-0.56	0.10
Baseline herbivory	0.02	0.14	-0.27	0.31
Time span	-0.28	0.16	-0.59	0.04
Site area (log)	0.28	0.16	-0.04	0.60

Supplementary Table 13: **Summary of the model testing the association between herbivory change and spp. exchange ratio (Figure 3b).** Parameter estimates, their standard error (SE) and 95% credible interval (CI). Both the response and predictor variables were scaled.

Predictor	Slope estimate	SE	lower 95% CI	upper 95% CI
Intercept	0.001	0.117	-0.228	0.232
Herbivore pressure	0.518	0.141	0.240	0.790
Baseline herbivory	0.036	0.122	-0.207	0.279
Time span	0.321	0.133	0.057	0.587
Site area (log)	-0.248	0.137	-0.510	0.023

Supplementary Table 14: **Summary of the model testing the association between changes in herbivory and species richness, after accounting for forest management change.** Parameter estimates, their standard error (SE) and 95% credible interval (CI). Both the response and predictor variables were scaled.

Predictor	Slope estimate	SE	lower 95% CI	upper 95% CI
Intercept	0.011	0.163	-0.315	0.327
Herbivore pressure	-0.223	0.173	-0.555	0.114
Baseline herbivory	0.022	0.148	-0.260	0.310
Time span	-0.270	0.162	-0.591	0.048
Site area (log)	0.275	0.167	-0.057	0.606
Mgmt change:decline (delta)	0.065	0.373	-0.662	0.810

Supplementary Table 15: **Summary of the model testing the association between changes in herbivory and species exchange ratio, after accounting for forest management change.** Parameter estimates, their standard error (SE) and 95% credible interval (CI). Both the response and predictor variables were scaled.

Predictor	Slope estimate	SE	lower 95% CI	upper 95% CI
Intercept	-0.046	0.133	-0.317	0.210
Herbivore pressure	0.495	0.146	0.207	0.783
Baseline herbivory	0.018	0.127	-0.234	0.268
Time span	0.308	0.136	0.045	0.578
Site area (log)	-0.251	0.141	-0.529	0.022
Mgmt change:decline (delta)	0.242	0.311	-0.373	0.855

Supplementary Table 16 - 23. *Model summaries for Figure 4: Herbivory and community composition.*

Supplementary Table 16: **Summary of the model testing the association between changes in herbivory and CWM N number (Figure 4a).** Parameter estimates, their standard error (SE) and 95% credible interval (CI). Both the response and predictor variables were scaled.

Predictor	Slope estimate	SE	lower 95% CI	upper 95% CI
Intercept	-0.001	0.117	-0.226	0.229
Herbivore pressure	0.432	0.149	0.138	0.725
Baseline herbivory	0.011	0.127	-0.228	0.264
Time span	0.162	0.137	-0.107	0.431
Site area (log)	0.116	0.144	-0.169	0.401

Supplementary Table 17: **Summary of the model testing the association between changes in herbivory and % non-native spp. (Figure 4c).** Parameter estimates, their standard error (SE) and 95% credible interval (CI). Both the response and predictor variables were scaled.

Predictor	Slope estimate	SE	lower 95% CI	upper 95% CI
Intercept	0.0025	0.1331	-0.2593	0.2614
Herbivore pressure	0.3665	0.1651	0.0452	0.6916
Baseline herbivory	-0.0868	0.1370	-0.3554	0.1816
Time span	0.0463	0.1523	-0.2549	0.3460
Site area (log)	0.0122	0.1591	-0.3047	0.3110

Supplementary Table 18: **Summary of the model testing the association between changes in herbivory and % red-listed spp. (Figure 4e).** Parameter estimates, their standard error (SE) and 95% credible interval (CI). Both the response and predictor variables were scaled.

Predictor	Slope estimate	SE	lower 95% CI	upper 95% CI
Intercept	0.0028	0.1408	-0.2854	0.2848
Herbivore pressure	-0.1929	0.1690	-0.5250	0.1367
Baseline herbivory	-0.0667	0.1458	-0.3545	0.2200
Time span	-0.0690	0.1534	-0.3742	0.2347
Site area (log)	-0.1379	0.1652	-0.4625	0.1841

Supplementary Table 19: **Summary of the model testing the association between changes in herbivory and % small-ranged spp. (Figure 4g).** Parameter estimates, their standard error (SE) and 95% credible interval (CI). Both the response and predictor variables were scaled.

Predictor	Slope estimate	SE	lower 95% CI	upper 95% CI
Intercept	0.0047	0.1359	-0.2564	0.2710
Herbivore pressure	-0.2463	0.1664	-0.5731	0.0860
Baseline herbivory	-0.1852	0.1471	-0.4743	0.1032
Time span	-0.0521	0.1608	-0.3692	0.2671
Site area (log)	-0.1261	0.1607	-0.4433	0.1823

Supplementary Table 20: **Summary of the model testing the association between changes in herbivory and CWM-N, after accounting for forest management change.** Parameter estimates, their standard error (SE) and 95% credible interval (CI). Both the response and predictor variables were scaled.

Predictor	Slope estimate	SE	lower 95% CI	upper 95% CI
Intercept	-0.123	0.134	-0.385	0.142
Herbivore pressure	0.376	0.142	0.102	0.660
Baseline herbivory	-0.035	0.124	-0.277	0.216
Time span	0.130	0.132	-0.130	0.389
Site area (log)	0.108	0.138	-0.168	0.379
Mgmt change:decline (delta)	0.579	0.315	-0.054	1.199

Supplementary Table 21: **Summary of the model testing the association between changes in herbivory and % non-native spp., after accounting for forest management change.** Parameter estimates, their standard error (SE) and 95% credible interval (CI). Both the response and predictor variables were scaled.

Predictor	Slope estimate	SE	lower 95% CI	upper 95% CI
Intercept	-0.077	0.155	-0.385	0.227
Herbivore pressure	0.331	0.170	-0.004	0.669
Baseline herbivory	-0.115	0.143	-0.386	0.174
Time span	0.022	0.153	-0.275	0.326
Site area (log)	0.004	0.163	-0.311	0.334
Mgmt change:decline (delta)	0.367	0.361	-0.341	1.079

Supplementary Table 22: **Summary of the model testing the association between changes in herbivory and % red-listed spp., after accounting for forest management change.** Parameter estimates, their standard error (SE) and 95% credible interval (CI). Both the response and predictor variables were scaled.

Predictor	Slope estimate	SE	lower 95% CI	upper 95% CI
Intercept	0.130	0.159	-0.177	0.432
Herbivore pressure	-0.134	0.174	-0.474	0.216
Baseline herbivory	-0.018	0.147	-0.306	0.274
Time span	-0.029	0.159	-0.339	0.288
Site area (log)	-0.130	0.162	-0.444	0.190
Mgmt change:decline (delta)	-0.614	0.367	-1.350	0.103

Supplementary Table 23: **Summary of the model testing the association between changes in herbivory and % small-ranged spp., after accounting for forest management change.** Parameter estimates, their standard error (SE) and 95% credible interval (CI). Both the response and predictor variables were scaled.

Predictor	Slope estimate	SE	lower 95% CI	upper 95% CI
Intercept	0.166	0.154	-0.143	0.467
Herbivore pressure	-0.174	0.166	-0.506	0.146
Baseline herbivory	-0.127	0.139	-0.400	0.142
Time span	-0.012	0.153	-0.312	0.286
Site area (log)	-0.112	0.156	-0.423	0.193
Mgmt change:decline (delta)	-0.736	0.356	-1.446	-0.030

Supplementary Table 24 - 25. *Model summaries for Supplementary Figure 2: Shrub cover versus non-native, nitrophilous spp.*

Supplementary Table 24: **Summary of the model testing the association between changes in shrub cover and % non-native spp.** Parameter estimates, their standard error (SE) and 95% credible interval (CI). Both the response and predictor variables were scaled.

Predictor	Slope estimate	SE	lower 95% CI	upper 95% CI
Intercept	-0.0106	0.1369	-0.2834	0.2594
Shrub cover	-0.3696	0.1393	-0.6367	-0.0913
Time span	0.1281	0.1482	-0.1595	0.4160
Site area (log)	0.0850	0.1533	-0.2121	0.3874

Supplementary Table 25: **Summary of the model testing the association between changes in shrub cover and CWM N number.** Parameter estimates, their standard error (SE) and 95% credible interval (CI). Both the response and predictor variables were scaled.

Predictor	Slope estimate	SE	lower 95% CI	upper 95% CI
Intercept	-0.0263	0.1256	-0.2715	0.2206
Shrub cover	-0.1939	0.1314	-0.4557	0.0590
Time span	0.2763	0.1409	-0.0126	0.5518
Site area (log)	0.2657	0.1423	-0.0147	0.5450

Supplementary Table 26 - 29. *Model summaries for Supplementary Figure 3: N-deposition and community composition.*

Supplementary Table 26: **Summary of the model testing the association between cumulative N-deposition and the %-change in red-listed spp. (Supplementary Figure 3a).** Parameter estimates, their standard error (SE) and 95% credible interval (CI). Both the response and predictor variables were scaled.

Predictor	Slope estimate	SE	lower 95% CI	upper 95% CI
Intercept	0.00097	0.13410	-0.25931	0.26405
N-dep	-0.45777	0.21303	-0.88617	-0.05272
Time span	0.16616	0.19624	-0.21195	0.55649
Site area (log)	-0.07654	0.15470	-0.38173	0.22986

Supplementary Table 27: **Summary of the model testing the association between cumulative N-deposition and the %-change in non-native spp. (Supplementary Figure 3c).** Parameter estimates, their standard error (SE) and 95% credible interval (CI). Both the response and predictor variables were scaled.

Predictor	Slope estimate	SE	lower 95% CI	upper 95% CI
Intercept	-0.0010	0.1307	-0.2593	0.2533
N-dep	0.5613	0.2041	0.1658	0.9601
Time span	-0.2072	0.1904	-0.5818	0.1691
Site area (log)	0.0053	0.1530	-0.2960	0.3019

Supplementary Table 28: **Summary of the model testing the association between cumulative N-deposition and the %-change in small-ranged spp. (Supplementary Figure 3b).** Parameter estimates, their standard error (SE) and 95% credible interval (CI). Both the response and predictor variables were scaled.

Predictor	Slope estimate	SE	lower 95% CI	upper 95% CI
Intercept	0.00341	0.13545	-0.26353	0.27257
N-dep	-0.37813	0.21241	-0.79709	0.04114
Time span	0.12171	0.20231	-0.28083	0.51614
Site area (log)	-0.09509	0.15906	-0.41251	0.21813

Supplementary Table 29: **Summary of the model testing the association between cumulative N-deposition and the change in CWM N-number. (Supplementary Figure 3d).** Parameter estimates, their standard error (SE) and 95% credible interval (CI). Both the response and predictor variables were scaled.

Predictor	Slope estimate	SE	lower 95% CI	upper 95% CI
Intercept	-0.0017	0.1314	-0.2593	0.2582
N-dep	0.1685	0.2081	-0.2512	0.5776
Time span	0.1702	0.1936	-0.2068	0.5472
Site area (log)	0.2327	0.1542	-0.0727	0.5366

Supplementary Table 30 - 37. *Model summaries for Figure 4: Interaction between herbivory and N-deposition.*

Supplementary Table 30: **Summary of model testing the interactive effect between herbivore pressure and N-deposition on CWM N-number. (Figure 4b).** Parameter estimates, their standard error (SE) and 95% credible interval (CI). Both the response and predictor variables were scaled.

Predictor	Slope estimate	SE	lower 95% CI	upper 95% CI
Intercept	-0.14346	0.13834	-0.41861	0.12963
Herbivore pressure	0.28525	0.15601	-0.01979	0.59231
N-dep	0.24089	0.19410	-0.14276	0.62363
Time span	0.10411	0.16984	-0.23513	0.44001
Site area (log)	0.12390	0.14429	-0.15784	0.41086
Herbivore:N-dep	0.32971	0.16528	0.00489	0.65462

Supplementary Table 31: **Summary of model testing the interactive effect between herbivore pressure and N-deposition on % non-native spp. (Figure 4d).** Parameter estimates, their standard error (SE) and 95% credible interval (CI). Both the response and predictor variables were scaled.

Predictor	Slope estimate	SE	lower 95% CI	upper 95% CI
Intercept	-0.15823	0.14254	-0.43938	0.12451
Herbivore pressure	0.19529	0.16362	-0.12837	0.51561
N-dep	0.66243	0.20276	0.26629	1.06086
Time span	-0.26308	0.17790	-0.61263	0.08861
Site area (log)	-0.07403	0.15048	-0.37599	0.21962
Herbivore:N-dep	0.36221	0.17277	0.02477	0.70093

Supplementary Table 32: **Summary of model testing the interactive effect between herbivore pressure and N-deposition on % red-listed spp. (Figure 4f).** Parameter estimates, their standard error (SE) and 95% credible interval (CI). Both the response and predictor variables were scaled.

Predictor	Slope estimate	SE	lower 95% CI	upper 95% CI
Intercept	0.16467	0.15405	-0.14038	0.46590
Herbivore pressure	0.01358	0.17541	-0.32855	0.36299
N-dep	-0.60393	0.21945	-1.03893	-0.17028
Time span	0.19537	0.19105	-0.18431	0.57162
Site area (log)	-0.06133	0.16282	-0.38160	0.26048
Herbivore:N-dep	-0.37769	0.18673	-0.74862	-0.00935

Supplementary Table 33: **Summary of model testing the interactive effect between herbivore pressure and N-deposition on % small-ranged spp. (Figure 4h).** Parameter estimates, their standard error (SE) and 95% credible interval (CI). Both the response and predictor variables were scaled.

Predictor	Slope estimate	SE	lower 95% CI	upper 95% CI
Intercept	0.22165	0.15068	-0.07421	0.51683
Herbivore pressure	0.03988	0.17192	-0.29525	0.38004
N-dep	-0.56607	0.21418	-0.98972	-0.14858
Time span	0.14544	0.18769	-0.22306	0.51553
Site area (log)	-0.08430	0.15878	-0.39849	0.22959
Herbivore:N-dep	-0.50683	0.18215	-0.86508	-0.14563

Supplementary Table 34: **Summary of model testing the interaction between changes in herbivory and N-deposition on CWM-N, after accounting for forest management change.** Parameter estimates, their standard error (SE) and 95% credible interval (CI). Both the response and predictor variables were scaled.

Predictor	Slope estimate	SE	lower 95% CI	upper 95% CI
Intercept	-0.261	0.151	-0.561	0.046
N-dep	0.113	0.205	-0.284	0.528
Herbivore pressure	0.234	0.163	-0.075	0.561
Baseline herbivory	-0.090	0.131	-0.351	0.168
Time span	0.148	0.174	-0.188	0.490
Site area (log)	0.142	0.144	-0.142	0.421
Mgmt change:decline (delta)	0.558	0.320	-0.084	1.186
Herbivore:N-dep	0.329	0.167	-0.003	0.655

Supplementary Table 35: **Summary of model testing the interaction between changes in herbivory and N-deposition on % non-native spp., after accounting for forest management change.** Parameter estimates, their standard error (SE) and 95% credible interval (CI). Both the response and predictor variables were scaled.

Predictor	Slope estimate	SE	lower 95% CI	upper 95% CI
Intercept	-0.185	0.163	-0.511	0.135
N-dep	0.631	0.222	0.192	1.068
Herbivore pressure	0.158	0.176	-0.193	0.502
Baseline herbivory	-0.089	0.141	-0.366	0.190
Time span	-0.237	0.185	-0.601	0.125
Site area (log)	-0.065	0.154	-0.368	0.240
Mgmt change:decline (delta)	0.075	0.347	-0.609	0.756
Herbivore:N-dep	0.386	0.182	0.032	0.748

Supplementary Table 36: **Summary of model testing the interaction between changes in herbivory and N-deposition on % red-listed spp., after accounting for forest management change.** Parameter estimates, their standard error (SE) and 95% credible interval (CI). Both the response and predictor variables were scaled.

Predictor	Slope estimate	SE	lower 95% CI	upper 95% CI
Intercept	0.230	0.173	-0.113	0.570
N-dep	-0.537	0.234	-0.997	-0.075
Herbivore pressure	0.019	0.184	-0.348	0.378
Baseline herbivory	-0.034	0.151	-0.326	0.263
Time span	0.186	0.196	-0.200	0.571
Site area (log)	-0.070	0.162	-0.388	0.250
Mgmt change:decline (delta)	0.358	0.362	-0.359	1.071
Herbivore:N-dep	-0.351	0.191	-0.728	0.025

Supplementary Table 37: **Summary of model testing the interaction between changes in herbivory and N-deposition on % small-ranged spp., after accounting for forest management change.** Parameter estimates, their standard error (SE) and 95% credible interval (CI). Both the response and predictor variables were scaled.

Predictor	Slope estimate	SE	lower 95% CI	upper 95% CI
Intercept	0.311	0.164	-0.012	0.632
N-dep	-0.484	0.225	-0.930	-0.045
Herbivore pressure	0.023	0.174	-0.316	0.368
Baseline herbivory	-0.111	0.142	-0.388	0.165
Time span	0.154	0.189	-0.215	0.527
Site area (log)	-0.090	0.155	-0.396	0.216
Mgmt change:decline (delta)	-0.544	0.350	-1.232	0.139
Herbivore:N-dep	-0.447	0.181	-0.804	-0.092

Supplementary Table 38 - 39. *Model summaries for Supplementary Figure 4: Differences in N-numbers.*

Supplementary Table 38: **Summary of the model testing for differences in N-number between threatened and non-threatened spp. (Supplementary Figure 4a).** Parameter estimates, their standard error (SE) and 95% credible interval (CI). Both the response and predictor variables were scaled.

Predictor	Slope estimate	SE	lower 95% CI	upper 95% CI
Intercept	5.34	0.09	5.17	5.51
Threatened - Yes	-1.41	0.17	-1.73	-1.08

Supplementary Table 39: **Summary of the model testing for differences in N-number between native and non-native species. (Supplementary Figure 4b).** Parameter estimates, their standard error (SE) and 95% credible interval (CI). Both the response and predictor variables were scaled.

Predictor	Slope estimate	SE	lower 95% CI	upper 95% CI
Intercept	6.52	0.12	6.29	6.75
Native - Yes	-1.25	0.10	-1.46	-1.05

Supplementary Table 40 - 43. *Model summaries for changes in herbivory versus community composition, accounting for productivity.*

Supplementary Table 40: **Summary of the model testing the association between changes in herbivore pressure and CWM-N, with productivity measured as AP:PET as a covariate.** Parameter estimates, their standard error (SE) and 95% credible interval (CI). Both the response and predictor variables were scaled.

Predictor	Slope estimate	SE	lower 95% CI	upper 95% CI
Intercept	0.000	0.127	-0.247	0.256
Herbivore pressure	0.420	0.151	0.117	0.722
Baseline herbivory	0.016	0.128	-0.235	0.269
Time span	0.173	0.148	-0.112	0.453
Site area (log)	0.119	0.146	-0.166	0.406
Productivity - AP:PET	0.032	0.136	-0.230	0.294

Supplementary Table 41: **Summary of the model testing the association between changes in herbivore pressure and % non-naitve spp., with productivity measured as AP:PET as a covariate.** Parameter estimates, their standard error (SE) and 95% credible interval (CI). Both the response and predictor variables were scaled.

Predictor	Slope estimate	SE	lower 95% CI	upper 95% CI
Intercept	0.001	0.134	-0.266	0.260
Herbivore pressure	0.348	0.173	0.011	0.688
Baseline herbivory	-0.078	0.146	-0.379	0.208
Time span	0.057	0.160	-0.265	0.368
Site area (log)	-0.001	0.161	-0.319	0.315
Productivity - AP:PET	0.059	0.150	-0.228	0.354

Supplementary Table 42: **Summary of the model testing the association between changes in herbivore pressure and % red-listed spp., with productivity measured as AP:PET as a covariate.** Parameter estimates, their standard error (SE) and 95% credible interval (CI). Both the response and predictor variables were scaled.

Predictor	Slope estimate	SE	lower 95% CI	upper 95% CI
Intercept	0.005	0.146	-0.286	0.287
Herbivore pressure	-0.187	0.181	-0.547	0.162
Baseline herbivory	-0.070	0.159	-0.384	0.244
Time span	-0.072	0.167	-0.396	0.254
Site area (log)	-0.107	0.167	-0.440	0.215
Productivity - AP:PET	-0.037	0.157	-0.349	0.263

Supplementary Table 43: **Summary of the model testing the association between changes in herbivore pressure and % small-ranged spp., with productivity measured as AP:PET as a covariate.** Parameter estimates, their standard error (SE) and 95% credible interval (CI). Both the response and predictor variables were scaled.

Predictor	Slope estimate	SE	lower 95% CI	upper 95% CI
Intercept	0.005	0.143	-0.275	0.280
Herbivore pressure	-0.239	0.176	-0.585	0.102
Baseline herbivory	-0.182	0.149	-0.464	0.108
Time span	-0.010	0.168	-0.343	0.315
Site area (log)	-0.127	0.169	-0.469	0.202
Productivity - AP:PET	0.057	0.160	-0.258	0.376

Supplementary Table 44 - 47. *Model summaries for changes in herbivory versus community composition, accounting for tree cover*

Supplementary Table 44: **Summary of the model testing the association between changes in herbivore pressure and CWM-N, with tree cover as a covariate.** Parameter estimates, their standard error (SE) and 95% credible interval (CI). Both the response and predictor variables were scaled.

Predictor	Slope estimate	SE	lower 95% CI	upper 95% CI
Intercept	-0.018	0.121	-0.252	0.218
Herbivore pressure	0.411	0.143	0.128	0.702
Baseline herbivory	0.033	0.130	-0.226	0.279
Time span	0.163	0.138	-0.114	0.428
Site area (log)	0.135	0.142	-0.148	0.414
Tree cover	0.172	0.127	-0.084	0.415

Supplementary Table 45: **Summary of the model testing the association between changes in herbivore pressure and % non-native spp., with tree cover as a covariate.** Parameter estimates, their standard error (SE) and 95% credible interval (CI). Both the response and predictor variables were scaled.

Predictor	Slope estimate	SE	lower 95% CI	upper 95% CI
Intercept	-0.005	0.140	-0.281	0.270
Herbivore pressure	0.353	0.168	0.020	0.685
Baseline herbivory	-0.118	0.153	-0.420	0.180
Time span	0.030	0.156	-0.281	0.336
Site area (log)	0.000	0.162	-0.326	0.316
Tree cover	0.000	0.140	-0.271	0.273

Supplementary Table 46: **Summary of the model testing the association between changes in herbivore pressure and % red-listed spp., with tree cover as a covariate.** Parameter estimates, their standard error (SE) and 95% credible interval (CI). Both the response and predictor variables were scaled.

Predictor	Slope estimate	SE	lower 95% CI	upper 95% CI
Intercept	0.018	0.143	-0.260	0.297
Herbivore pressure	-0.207	0.174	-0.557	0.125
Baseline herbivory	-0.128	0.166	-0.461	0.201
Time span	-0.088	0.160	-0.406	0.235
Site area (log)	-0.176	0.169	-0.508	0.154
Tree cover	0.023	0.150	-0.282	0.312

Supplementary Table 47: **Summary of the model testing the association between changes in herbivore pressure and % non-native spp., with tree cover as a covariate.** Parameter estimates, their standard error (SE) and 95% credible interval (CI). Both the response and predictor variables were scaled.

Predictor	Slope estimate	SE	lower 95% CI	upper 95% CI
Intercept	0.020	0.141	-0.258	0.296
Herbivore pressure	-0.241	0.173	-0.584	0.090
Baseline herbivory	-0.205	0.161	-0.533	0.122
Time span	-0.059	0.159	-0.382	0.254
Site area (log)	-0.137	0.169	-0.468	0.196
Tree cover	-0.066	0.146	-0.349	0.216

Supplementary Table 48 - 51. *Model summaries for changes in herbivory versus community composition, excluding baseline herbivory.*

Supplementary Table 48: **Summary of the model testing the association between changes in herbivore pressure and CWM-N, excluding baseline herbivory.** Parameter estimates, their standard error (SE) and 95% credible interval (CI). Both the response and predictor variables were scaled.

Predictor	Slope estimate	SE	lower 95% CI	upper 95% CI
Intercept	0.001	0.117	-0.227	0.231
Herbivore pressure	0.430	0.145	0.142	0.715
Time span	0.163	0.134	-0.104	0.433
Site area (log)	0.114	0.139	-0.167	0.392

Supplementary Table 49: **Summary of the model testing the association between changes in herbivore pressure and % non-native spp., excluding baseline herbivory.** Parameter estimates, their standard error (SE) and 95% credible interval (CI). Both the response and predictor variables were scaled.

Predictor	Slope estimate	SE	lower 95% CI	upper 95% CI
Intercept	-0.001	0.133	-0.258	0.259
Herbivore pressure	0.387	0.162	0.078	0.715
Time span	0.036	0.152	-0.262	0.336
Site area (log)	0.015	0.156	-0.287	0.315

Supplementary Table 50: **Summary of the model testing the association between changes in herbivore pressure and % red-listed spp., excluding baseline herbivory.** Parameter estimates, their standard error (SE) and 95% credible interval (CI). Both the response and predictor variables were scaled.

Predictor	Slope estimate	SE	lower 95% CI	upper 95% CI
Intercept	-0.003	0.141	-0.279	0.284
Herbivore pressure	-0.177	0.169	-0.508	0.168
Time span	-0.066	0.158	-0.374	0.255
Site area (log)	-0.135	0.167	-0.469	0.184

Supplementary Table 51: **Summary of the model testing the association between changes in herbivore pressure and CWM % small-ranged spp., excluding baseline herbivory.** Parameter estimates, their standard error (SE) and 95% credible interval (CI). Both the response and predictor variables were scaled.

Predictor	Slope estimate	SE	lower 95% CI	upper 95% CI
Intercept	0.001	0.140	-0.278	0.274
Herbivore pressure	-0.193	0.171	-0.531	0.139
Time span	-0.062	0.160	-0.371	0.259
Site area (log)	-0.122	0.164	-0.448	0.203
